# Understanding everyday victimization experiences in vulnerable youth: an ecological momentary assessment approach

**DOI:** 10.1007/s00787-025-02829-z

**Published:** 2025-07-31

**Authors:** Sophie Charlotte Niestroj, Marco Giurgiu, Maren Boecker, Sarah Steden, Lisa Knobloch, Ann-Katrin Wiemann, Arnold Lohaus, Ulrich W. Ebner-Priemer, Kerstin Konrad

**Affiliations:** 1 Child Neuropsychology Section, Department of Child and Adolescent Psychiatry, Uniklinik RWTH Aachen, Neuenhofer Weg 21, 52074 Aachen, Germany; 2 Mental mHealth Lab, Institute of Sports and Sport Sciences, Karlsruhe Institute of Technology, Karlsruhe, Germany; 3 Faculty of Psychology and Sports Science, Bielefeld University, P.O. Box 10 01 31, 33501 Bielefeld, Germany; 4 JARA-Brain Institute II, Molecular Neuroscience and Neuroimaging, RWTH Aachen & Research Centre Juelich, Juelich, Germany; 5 Department of Psychiatry and Psychotherapy, Central Institute of Mental Health, University of Heidelberg, Medical Faculty Mannheim, Mannheim, Germany

**Keywords:** EMA, (re-)victimization, Out-of-home care, Psychopathology, Affective well-being

## Abstract

Early victimization significantly increases the risk of subsequent revictimization throughout life, yet the underlying psychological mechanisms remain poorly understood. Ecological Momentary Assessment (EMA) studies hold promise in clarifying how contextual factors and social attributions influence everyday victimization experiences. This study utilized EMA to examine daily victimization in out-of-home care (OOHC), a high-risk group for (re-)victimization, compared to children and adolescents living with their biological families (BF). Over two weeks, 157 participants, aged eight to 21 years (*N* = 68 in OOHC and *N* = 89 in BF group) reported their victimization experiences, including contextual and individual factors via EMA. Additionally, participants answered standardized questionnaires on lifetime victimization and psychopathology. Attending school was identified as a contextual risk factor for everyday victimization experiences. Higher levels of psychopathology and OOHC residence were associated with increased everyday victimization. In contrast, lifetime victimization did not predict current victimization frequency but significantly impacted daily affective states and social acceptance levels. Internal attribution styles for everyday victimization experiences were associated with lower affective valence and social acceptance. These findings emphasize the heightened vulnerability of children and youth in OOHC to frequent victimization, with internal attribution styles in response to victimization being a significant risk factor for negative affect and feelings of social ostracism. In conclusion, this study sheds light on the nature of victimization experiences among OOHC and BF. It emphasizes the importance of understanding and addressing internal attribution styles in interventions designed to support vulnerable populations like children and adolescents in OOHC.

**Supplementary Information:**

The online version contains supplementary material available at 10.1007/s00787-025-02829-z.

## Introduction

Worldwide, approximately 2.4 million children and adolescents live in residential care settings (UNICEF [1]). Youth welfare services, including foster care, residential care, and adoption services, provide essential support to children who have been maltreated or whose parents are unable to provide adequate care. However, it has been shown that the majority of these children have not only experienced neglect and abuse in the past but also continue to face an increased risk of various forms of subsequent victimization over their entire life span [2–5]. Such revictimization experiences may range from daily experiences of social exclusion or direct verbal attacks by peers and siblings [6], to dating violence or other severe forms of sexual or physical maltreatment by peers or adult caregivers [7, 8]. Poly-victimization has tremendous and long-lasting effects on mental health, and developmental outcomes [9]. So far, neither the psychological mechanisms underlying the vicious cycle of early and later victimization experiences nor the environmental risk contexts in which later victimization experiences occur have been fully explored.

To understand the mechanisms sustaining this cycle of victimization, Bockers and Knaevelsrud [10] proposed a bio-psycho-social vulnerability model. This model suggests that early trauma, common among children in out-of-home care (OOHC), disrupts the development of stress regulation systems, leading to heightened physiological arousal and impaired emotion regulation. Such dysregulation may predispose individuals to misinterpret social cues and react with heightened emotional intensity, potentially increasing their susceptibility to further victimization.

From a cognitive perspective, attribution theory adds an essential layer to this model. The way individuals interpret and assign meaning to adverse experiences, whether through internal ("It’s my fault") or external ("Others are unfair") attributions, significantly shapes their emotional responses, behavior, and interactions with others [11]. In the context of peer victimization, especially stable, internal attributions have been linked to increased psychological distress and a higher likelihood of revictimization [12]. Thus, attributional style may not only reflect past negative experiences but also contribute to a persistent cycle of victimization, particularly in vulnerable populations.

Importantly, environmental context also plays a critical role in shaping exposure to risk. Using ecological momentary assessment (EMA) Yoon et al. [13] reported that adolescents with more maltreatment experiences were exposed to more violent and risky activity spaces. Similarly, Magallón-Neri et al. [14] showed that high victimization correlated with more time away from home, more time at sports clubs, and emotional and behavioral problems in adolescents. These settings interact with emotional and behavioral vulnerabilities to exacerbate risk. EMA enables the real-time capture of emotional states and social experiences in natural contexts, offering a powerful tool to examine the micro-dynamics of victimization and affect regulation in the context of daily life [15].

While the long-term consequences of childhood maltreatment on the development of psychopathology are well-documented, recent research is beginning to shed light on its impact on everyday emotional experiences. Specifically, findings indicate that individuals with higher levels of childhood maltreatment experience more negative emotions in their daily lives [16]. Gaining a better understanding of these, day-to-day affective dynamics is essential, as they may play a critical role in the underlying mechanisms of revictimization.

Social exclusion, in particular, poses a serious threat to a child’s fundamental need to belong, which is essential for healthy emotional and social development. It has emerged as a key psychosocial factor contributing to both the onset and maintenance of mental health difficulties [17]. Children in OOHC who are often stigmatized and demonstrate poor social skills [18–20], might face an increased risk of exclusion (ostracism) in peer contexts. Notably, studies show that adolescents in OOHC exhibit heightened neural sensitivity to social exclusion [17, 21], which may not only increase vulnerability to psychopathology but also to further victimization, highlighting the need to investigate how victimization shapes emotional and social functioning in daily life.

Taken together, these findings suggest that revictimization in OOHC populations arises from a complex interplay of emotional, cognitive, and contextual factors. Thus, the current study aimed to use EMA diaries to specifically explore the relationship between everyday victimization experiences and the contexts in which they occur, how they are perceived and interpreted and relate them to lifetime victimization and psychopathology in high and low-risk participants for early childhood maltreatment.

We hypothesized that participants in OOHC experience both more lifetime and everyday victimization compared to participants living with their biological families (BF) (H1) and that both, higher levels of lifetime victimization experiences and psychopathology are positively associated with a greater number of everyday victimization experiences (H2). Furthermore, we expected that lifetime victimization would negatively impact affective states and feelings of social acceptance in daily life (H3).

## Methods

The study was part of a multicenter project, funded by the German Federal Ministry of Education and Research (BMBF). *N* = 118 (OOHC) and *N* = 134 living with their BF took part in an online study, in which various risk and protective factors as well as victimization experiences and psychopathology were assessed via self- and other reports (see Wiemann et al., [5]). A subsample of those participants (N = 68 OOHC and N = 89 BF), consisting of children and adolescents aged 10 years and older, additionally participated in the current EMA study. Participants in OOHC had been living with their social family or in residential care for at least six months. Recruitment took place via child welfare agencies, residential care facilities, foster care networks, schools and social media. The study was approved by local Ethics Committees of the coordinating sites and was conducted according to the declaration of Helsinki. Informed consent was received from all participants and legal guardians. Based on the guidelines proposed by Arend and Schäfer [22]regarding minimum detectable effect sizes (MDES), the current dataset is sufficiently powered to detect small effects in within-subject associations and moderate to large effects in between-subject comparisons.

### Assessment instruments

#### Juvenile victimization questionnaire (JVQ-R2)

The revised German translation of the Lifetime Screener Sum Version of the Juvenile Victimization Questionnaire was used [23]. The Screener Sum Version of the JVQ-R2 consists of 34 items, organized into five modules: Conventional Crimes, Child Maltreatment, Peer and Sibling Victimization, Sexual Victimization, and Witnessing and Indirect Victimization. In alignment with German law, Item 10 and Item 28 (Physical Abuse by Caregiver and Witness to Parent Assault on Sibling) were modified to include spanking as a form of child maltreatment. Item 26 (Statutory Rape and Sexual Misconduct) was excluded due to the age range of the participants, resulting in a total of 33 items. For each item, participants indicated on a dichotomous scale (yes/no) whether they had experienced the described form of victimization during their lifetime. Module scores were only calculated if no more than one item per module was missing. The internal consistency in the current study shows a Cronbach's alpha of 0.87 for the youth’s self-report and of 0.85 for the caregiver report. Further analysis was conducted using the parental version.

#### Child behavior check list (CBCL) and youth self-report (YSR)

The German translation of the CBCL by Döpfner et. al., [24] was assessed by parents for children and adolescents aged 8 to 21 years with 118 items, and the YSR was completed by children and adolescents aged 11 to 21 years with 118 items. In this study, the two suicide items were excluded. Three superior scales can be calculated for the CBCL/YSR (internal problems, external problems, total). Items are judged on a three-point scale: “not true” (0), “somewhat true” (1), or “always or often true” (2). Nationally representative age- and gender related norm data (T-values) for the CBCL and YSR were used. Internal consistencies for internalizing (Cronbach’s alpha = 0.84), for externalizing problems (Cronbach’s alpha = 0.89) and the CBCL total score (Cronbach’s alpha = 0.98) were good to excellent in the current sample.

#### Ecological momentary assessments (EMA)

All participants received a study smartphone with the MovisensXS app (Movisens GmbH, Karlsruhe, Germany), enabling the smartphones to function as electronic diaries. Prompts were triggered over a total period of 14 days (including 64 momentary assessments in total). During school days, prompts were scheduled randomly four times/day and six times/day on weekends. At each prompt, participants were asked to indicate whether any of the 14 possible victimization experiences had occurred since the last assessment, using a yes/no rating scale. The 14 possible victimization experiences were categorized into four groups: physical, relational, cyber, and parental victimization experiences (see supplementary material Table 4). Physical victimization included experiences such as being forced to do something against one's will, being harassed, hit, pushed, or physically attacked, and having belongings stolen or intentionally damaged. Relational victimization involved instances of deliberate exclusion, spreading of lies or rumors, being ignored, losing friendships, or being ridiculed. Cyber victimization encompassed private messages being shared without consent, unauthorized photos posted online, or receiving threatening or aggressive messages. Lastly, parental victimization involved feeling unloved or unsupported by one's parents. Additionally, participants were asked to indicate if they had engaged in any of the same victimization behaviors towards someone else to assess offending behaviors. All victimization and offending questions were coded as binary (0 = No, 1 = Yes), and participants were asked to indicate whether each event had occurred by responding accordingly. For each reported victimization, follow-up questions were asked regarding the context, including the perpetrator, the location of the incident and the attribution of the victimization experience (internal stable, internal unstable, external). The location and perpetrator were assessed using a forced-choice format. Participants were presented with a predefined list of possible response options and asked to select the one that fit the situation. See supplementary material Figs. 4, 5, and 6 for an overview of all possible responses. Regarding attribution, internal stable attributions refer to a person’s belief that the cause of an event lies within themselves and is consistent over time. Internal unstable attributions reflect the belief that the cause is internal but temporary. In contrast, external attributions indicate that the cause is perceived as originating outside the individual, such as from situational factors or the behavior of others.

Furthermore, current affective states were measured at each prompt by a short scale comprising two bipolar items for the mood dimensions valence (items: unwell to well, discontent to content), energetic arousal (items: no energy to total energy, tired to awake), and calmness (items: tense to relaxed, agitated to calm), respectively. The instrument was based on the Multidimensional Mood Questionnaire [25] for ambulatory assessment studies. Findings indicate that using a three-dimensional model, encompassing valence, energetic arousal, and calmness, is highly sensitive in detecting within-person changes in affective states over time [25]. The two items for each affective state were averaged for further analysis. Within-person reliability coefficients, calculated using the Spearman-Brown correction, were 0.64 for the valence rating, 0.68 for energetic arousal, and 0.64 for calmness. We added one additional item capturing the feeling of social acceptance (inclusion vs. exclusion). At the end of each EMA assessment, participants were asked on a 100-point Likert scale how honestly they had answered the questions and how motivated they had been to answer them. An average motivation and honesty score was calculated per person. All responses were automatically time-stamped, and data were assessed, stored, and uploaded anonymized and encrypted on the MovisensXS servers. Technical problems were rare and could be reported by the participants via the text messaging system.

#### Statistical analyses

First, we inspected descriptive statistics and group comparisons between participants in OOHC and BF for sociodemographic variables, psychopathology and compliance of EMA. Next, for each of the 14 potential victimization experiences, we calculated frequency, percentage, and cumulative percentage. We conducted multiple comparisons using chi-square tests (χ^2^) to determine whether participants in OOHC experienced more victimization in everyday life compared to participants in BF (Hypothesis 1). We applied Benjamini–Hochberg False Discovery Rate (FDR) corrections to control for multiple testing. In accordance with ASEBA's official scoring instructions for the CBCL and YSR, the Expectation–Maximization (EM) algorithm was applied to impute data solely for cases with fewer than eight missing values. The Little’s test confirmed that the data were completely missing at random (MCAR). For participants missing more than eight values, data was imputed as follows. The dataset was prepared for imputation by first calculating scores for various scales and creating dummy variables to track missing data. To assess randomness, the extent and patterns of missing data were examined using visualization techniques, chi-square tests, and t-tests. A regression analysis was used to predict missing data, and the Multivariate Imputation by Chained Equations (MICE) package by Stef van Buuren (2020) was used to impute missing values. Imputed datasets were generated, and the robustness of the model was cross-validated, achieving a Root Mean Square Error (RMSE) of 3.06, indicating acceptable prediction accuracy. Effect sizes (Cohen's d) were calculated in R-Studio using the lsr package by Navarro (2022) version 0.5.2. Based on the benchmarks suggested by Cohan [26] effect sizes of at least *d* = 0.2 are considered as small, *d* = 0.5 as medium, and *d* = 0.08 as large.

To gain a better understanding of potential underlying mechanisms, we investigated contextual factors for relational and physical victimization experiences, as well as individual factors for relational victimization experiences. To assess whether lifetime victimization experiences and psychopathology influence everyday victimization experiences, we conducted multiple regression analyses (H2).

Regarding the EMA data, we conducted multilevel analyses to examine the effects of lifetime and current victimization experiences and attribution style on affective states (valence, arousal, calmness) and feelings of social acceptance: Y(valence, arousal, calmness, social acceptance)_ij_ = age_j_ + sex_j_ + group_j_ + lifetime victimization_j_ + psychopathology_j_ + attribution of everyday victimization experience_ij_ + school-day_ij_ + location_ij_ (H3). We calculated a random intercept random slope model with repeated measurements (level 1) nested within participants (level 2) for each outcome. First, intraclass correlations (ICCs) were estimated using unconditional models, including ratings of affective state dimensions and feelings of social acceptance as outcomes. Second, we added the predictors age [yrs], sex [male vs. female], group [BF vs. OOHC], lifetime victimization [JVQ], psychopathology [CBCL total], attribution of current victimization experiences [internal stable; internal unstable; external] and day [weekday vs weekend] and location [school; work; home; on the way; outside; hobby; somewhere else] to our models. We included significant (p < 0.05) random effects for each predictor. In the interest of model parsimony, nonsignificant random effects were deleted, resulting in different models. The analyses were performed using IBM SPSS Statistics 24 (IMB corp., Armonk, NY, USA).

As the prevalence of self-reported offending behavior was very low (< 6% of all prompts), we focused on victimization experiences only. However, for transparency, we report the assessed offending behaviors, although we do not interpret them.

## Results

### Sociodemographic and psychopathology

No group differences in subjects’ mean age and parental level of education were found between OOHC and BF (See Table 1). However, the average age of caregivers was significantly higher in parents of OOHC (*M* = 50.5, *SD* = 6.6) compared to BF (*M* = 46.7, *SD* = 5.5). Participants in OOHC showed significantly more externalizing, internalizing, and total psychopathology (CBCL) compared to BF (See Table 1). The average externalizing (*M* = 62.3, *SD* = 11.6) and total psychopathology (*M* = 65.9, *SD* = 9.1) T-scores in OOHC fall within the clinical range.

**Table 1:** Descriptive statistics and group comparisons

Variable	OOHC (*N* = 68)	BF (89)	Sig	Cohens d
Sociodemographics				
Age Child (M/SD)	13.3/2.7	13.7/3.4	.42	.13
Age Participating Parent (M/SD)	50.5/6.6	46.7/5.5	<.001**	.64
Gender Child (%_female_/%_male/_%_na_)	(%47.1/%47.1_/_%5.8)	(%55.1/%39.3_/_%5.6)	0.07 (X^2^)	-
Parents level of Education (M/SD)	7.3/3.3	6.9/3.2	.51	.11
Psychopathology				
CBCL External (M _*t*-value_/SD)	62.3/11.6	47.6/9.2	<.001**	1.41
CBCL Internal (M _*t*-value_/SD)	58.4/11.4	49.6/9.2	<.001**	.86
CBCL Total (M _*t*-value_/SD)	65.9/9.1	47.9/8.2	<.001**	2.09
Victimization				
JVQ Total (self-rating)	10.5/5.2	7.8/4.6	<.001**	.60
JVQ Total (parent rating)	9.8/4.2	5.6/2.9	<.001**	1.21
Ecological Momentary Assessments (EMA)				
EMA Victimization (%)	15.5%	6%	.07 (X^2^)	-
EMA Offending (%)	8%	3%	.29 (X^2^)	-
EMA attribution (frequency & percentage) _(internal stable/internal unstable/external/no victimization)_	44/28/37/7214 .6%/.4%/.5%/98.5	13/15/16/11065 .1%/.1%/.1%/99.6%	<.001** (X^2^)	-
EMA Compliance (M/SD)	55.9/23.9	64.9/21.5	.01*	.39
EMA Honesty	95.3/5.8	93.2/11.9	.05*	.20
EMA Motivation	64.4/25.3	60.6/24.9	.27	.15
EMA Valence	78.9/14.9	78.9/15.4	.97	-.01
EMA Calmness	75.7/15.4	76.4/16.3	.78	-.05
EMA Energetic arousal	59.5/22.2	65.9/17.2	.06	-.34
EMA Social acceptance	83.9/16.8	86.9/15.3	.26	-.14

Results include all points in time; *M* Mean, *SD* Standard Deviation, *(X2) * based on X2 test, *Sig* Significance*indicates *p* < 0.05; **indicates *p* < 0.01. *CBCL* Child Behavior Checklist, *JVQ* Juvenile Victimization Questionnaire

### Validation of victimization experiences as assessed by EMA and by questionnaires

Victimization experiences assessed via the JVQ showed significantly more victimization experiences in OOHC compared to subjects living with their BF (See Table 1). The largest differences between both groups were found for the parent-rated assessment scales and the smallest for EMA ratings. Additionally, EMA ratings of participants in OOHC showed a significantly higher rate of involvement in offending behaviors (8%) compared to participants living with their biological families (BF) (3%). Lifetime victimization experiences (JVQ) and current victimization (EMA) in the self-ratings showed no correlation, *r*(155) = 0.08, *p* = 0.30.

### EMA attrition, compliance, motivation and honesty

Among the 114 children and adolescents living with their BF, 92 smartphones were sent out. Twenty-two participants were either too young or declined to participate in the EMA part of the study and therefore did not receive a device. Of those who received a smartphone, only three participants dropped out during the 14-day EMA phase, resulting in 89 participants from the BF group who completed the full assessment period. In the OOHC group (*n* = 114), a total of 76 smartphones were sent out. Thirty-eight participants were either too young or declined to participate in EMA and thus did not receive a smartphone. Among the 76 participants who received a smartphone, five never started the assessment, and three dropped out during the EMA phase. Consequently, 68 participants in the OOHC group completed the full 14-day EMA assessment. A total of 18.829 prompts were sent, out of which 5.350 e-diary prompts were completed. The 142 participants answered a range of 3–168 prompts. Participants with < 30% responses to the e‐diary prompts were excluded from further analysis (*N* = 15). The average EMA compliance for 14 days was higher in the BF group (*M* = 64.9, *SD* = 21.5) than in the OOHC group (*M* = 55.9, *SD* = 23.9, *t*(139) = −2.5, *p* = 0.01). The mean motivation of answering the EMA questions was comparable between participants in OOHC (*M* = 64.4, *SD* = 25.3) and in BF (*M* = 60.6, *SD* = 24.9, *t*(152) = 1.11, *p* = 0.27). The data regarding honesty were excellent. Participants in OOHC reported having answered the EMA questions slightly more honestly (*M* = 95.3, *SD* = 5.8) compared to those living with their BF (*M* = 93.2, *SD* = 11.9, *t*(256) = 1.95, *p* = 0.05).

**Fig. 1:**
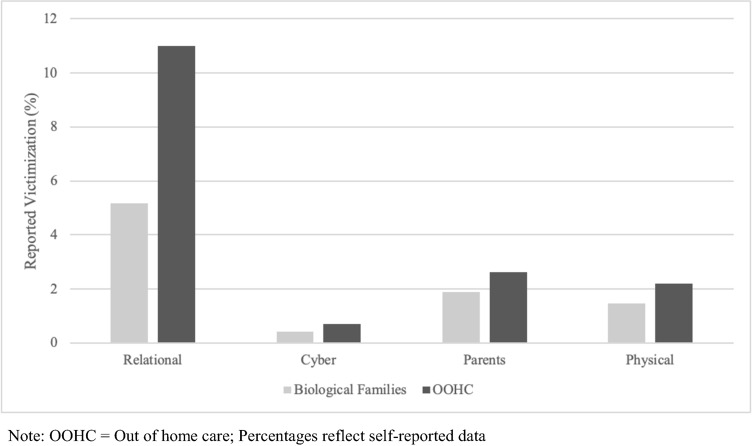
EMA victimization experiences (%)

The overall distribution of victimization experiences assessed via EMA can be seen in Fig. 1. Due to low overall counts of everyday victimization experiences over two weeks, we investigated contextual factors for relational and physical victimization experiences, and individual factors for relational victimization experiences, as they showed most responses.

### Everyday victimization experiences assessed via EMA (H1)

**Fig. 2:**
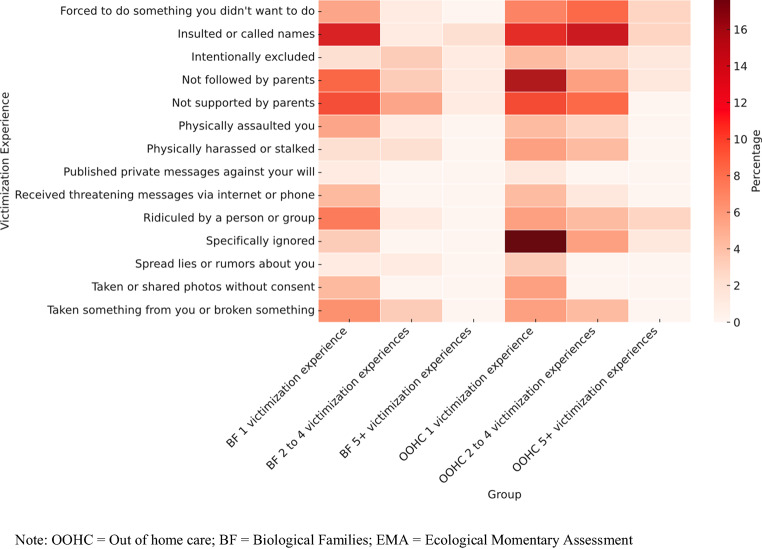
Heatmap of EMA Victimization experiences

Participants in OOHC reported a higher probability of total victimization experiences via EMA (*M* = 16%) compared to those living with their biological families (*M* = 6%). However, this difference did not reach statistical significance (*X*^*2*^(1,157) = 3.15, *p* = 0.07). To allow for testing of equality of proportions across each of the 14 possible victimization types, the reported frequencies were categorized into four groups (0, 1, 2–4, and ≥ 5 victimization experiences). For each of these victimization experiences, frequency, percentage, cumulative percentage, and group comparisons are reported (see supplementary material Table 4) and illustrated in Fig. 2. Although numerically, participants in OOHC reported a higher frequency of several victimization experiences, only one victimization experience differed significantly in frequency between the groups, after applying Benjamini–Hochberg False Discovery Rate (FDR) corrections: Experience 6 (“someone specifically ignored you or no longer wanted to be friends with you” (*X*^*2*^(2,157) = 12.2, *p* = 0.00) was more prevalent in the OOHC group. No significant differences in proportions were found for the other victimization experiences.

### Reported location of victimization experiences (EMA)

Participants in both OOHC and those living with their BF most commonly reported experiencing victimization at school. Specifically, 26% in OOHC and 28% participants in BF reported physical victimization occurring at school. Additionally, 23% of the BF and 22% of the OOHC reported physical victimization happened at home. For relational victimization, 62% of participants in OOHC and 51% of participants in BF indicated that it happened at school. Percentages of relational and physical victimization experiences occurring elsewhere can be found in the supplementary material (see Figs. 4 and 5). Missing data for each group were included separately in the graphs.

### Reported perpetrators of relational victimization experiences (EMA)

Overall counts of victimization experiences for cyber, parental, and physical victimization were sparse. Therefore, only data on reported perpetrators of relational victimization are presented. In both groups, classmates were most often identified as perpetrators (BF = 36%; OOHC = 19%), followed by friends (BF = 17%; OOHC = 13%). Subjects in OOHC had 50% missing data, more than double the amount of missing data compared to subjects living with their biological families (23%). An overview of all reported perpetrators in each group can be found in the supplementary material (see Fig. 6).

### Effects of lifetime victimization experiences and psychopathology on everyday victimization experiences (H2)

A multiple regression analysis was conducted to examine the relationship between everyday victimization experiences and the predictor variables: psychopathology, lifetime victimization, sex, age, group (OOHC vs. BC), location, and school day. The overall model was statistically significant, *F*(7, 128) = 2.72, *p* = 0.012, and explained 13.6% of the variance in everyday victimization experiences (*adjusted R*^*2*^ = 0.086). Group (OOHC vs. BF) emerged as a significant predictor of everyday victimization (*β* = 0.250, t = 2.114, *p* = 0.037), with participants from the OOHC group reporting more everyday victimization experiences compared to the BF group. Psychopathology was also a significant predictor (β = 0.235, t = 1.982, *p* = 0.050), indicating that higher psychopathology scores were associated with increased everyday victimization. Contrary to our initial hypotheses (H2), lifetime victimization experiences did not predict everyday victimization experiences (*β* = −0.162, *t* = −1.524, *p* = 0.128). Neither did sex, age, location, or school day (See Table 2).

**Table 2:** Multiple regression analysis to predict everyday victimization experiences

Outcome	Predictor	β-coefficient	Standardized β-coefficient	SE	*t*-value	*p*-value
Everyday victimization	sex	0.859	0.065	1.141	0.753	0.453
	group	2.102	0.250	0.994	2.114	0.037
	age	0.044	0.068	0.057	−0.765	0.445
	lifetime victimization	0.157	0.162	0.102	−1.534	0.128
	psychopathology	0.084	0.235	0.042	1.982	0.050
	school day	−0.003	0.025	0.010	−0.285	0.776
	location	−0.29	−0.101	0.025	−1.171	0.244

R2 0.136, Adjusted R2=0.086. Overall model: F(7, 128) =2.72, p 0.012

### Effects of victimization experiences on everyday affective states (valence, energetic arousal, calmness) and social acceptance (H3)

#### Momentary valence

**Fig. 3:**
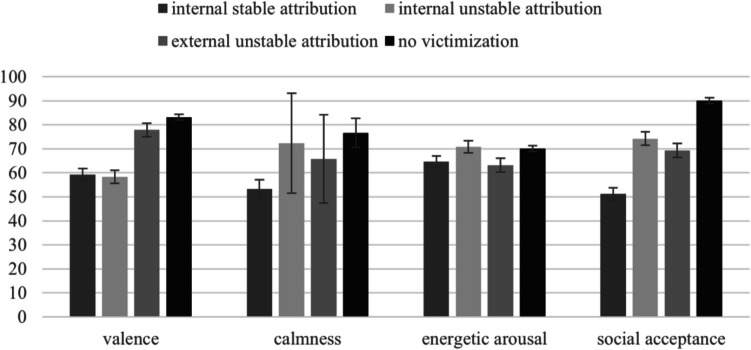
EM-means for all affective states and social acceptance

Within-subject analysis revealed that lifetime victimization, group (OOHC or BF), and attributions for victimization significantly predicted momentary valence. In specific, experiences of being victimized over one's lifetime significantly predicted momentary valence (*std. β* = −0.937, *p* = 0.029). Here, the lifetime victimization experience was associated with a reduction in momentary affective valence by 0.9 points. Moreover, the group variable (OOHC or BF) significantly predicted momentary valence. Participants living in OOHC had a reduction of valence by 0.11 points (std. *β* = −10.70, *p* = 0.01). Valence was significantly affected by internal attributions for current victimization experiences. Specifically, participants who made internal stable attributions showed a decrease in valence (*std. β* = −23.803, *p* < 0.001) compared to those with no victimization, and a similar decrease was observed for those with internal unstable attributions (*std. β* = −24.728, *p* < 0.001). However, no significant difference was found between no victimization and external unstable attributions, indicating that valence only decreased when victimization was attributed internal. No significant effects were found for age, sex, location, psychopathology, and school day. Significant random effects were found for attribution of everyday victimization (*p* < 0.001) and school day (*p* < 0.001), indicating variability between participants. Figure 3 displays the estimated marginal means for each attribution style and pairwise comparisons of the simple main effects between all attribution styles. These pairwise comparisons are based on estimated marginal means derived from our model and serve as a descriptive, model-based supplement to the main fixed effects reported in Table 3. While not equivalent to traditional ANOVA post-hoc tests, this approach allows for more precise interpretation. Notably, pairwise comparisons revealed that those with an internal stable attribution reported significantly lower valence than those with an external unstable attribution (*p* = 0.01). Furthermore, internal unstable attribution showed lower valence than external unstable attribution (*p* = 0.01). No difference was found between internal stable and internal unstable attribution (*p* = 0.89).

**Table 3:** Multilevel analyses to predict everyday affective states and social acceptance: fixed and random effects

Fixed effects							Random effects			
Model	Predictor	β-coefficient	SE	*t*-value	df	*p*-value	Variance estimate	SE	Wald-Z	*p*-value
Model: Valence	Intercept	102.786	16.743	6.139	121.399	< 0.001	254.142	6.709	37.879	< 0.001
	sex (0)	−1.348	4.565	−0.295	115.351	0.768				
	group (0)	−10.702	4.065	−2.633	117.543	0.010				
	age	−0.142	0.237	−0.599	120.269	0.0551				
	attribution-internal stable^1^	−23.803	4.807	−4.952	133.316	< 0.001				
	attribution-external^1^	−5.217	5.160	−1.011	148.350	0.314				
	attribution-internal unstable^1^	−24.728	5.576	−4.435	164.791	< 0.001				
	lifetime victimization (JVQ)	−0.937	0.423	−2.214	118.984	0.029				
	psychopathology	−0.060	0.175	−0.344	119.746	0.731				
	school day	−0.303	1.293	−0.234	72.594	0.816				
	location	1.732	0.933	1.857	3037.392	0.063				
Model: Energetic Arousal	Intercept	85.184	17.724	4.806	119.045	< 0.001	442.377	11.524	38.383	< 0.001
	sex (0)	0.830	4.919	0.169	116.675	0.866				
	group (0)	−5.618	4.323	−1.299	116.687	0.196				
	age	−0.268	0.248	−1.082	116.408	0.282				
	attribution-internal stable^1^	−5.391	4.053	−1.330	3099.346	0.184				
	attribution-external^1^	0.717	4.449	0.161	3057.011	0.872				
	attribution-internal unstable^1^	−6.938	5.154	−1.346	3001.132	0.178				
	lifetime victimization (JVQ)	−0.690	0.449	−1.556	119.638	0.122				
	psychopathology	0.013	0.185	0.075	118.881	0.940				
	school day	0.330	1.057	0.312	3116.435	0.755				
	location	4.994	1.671	2.989	81.192	0.004				
Model: Calmness	Intercept	96.186	16.432	5.853	121.782	< 0.001	283.785	7.446	38.112	< 0.001
	sex (0)	5.019	4.507	1.114	116.209	0.268				
	group (0)	−10.185	3.997	−2.548	118.302	0.012				
	age	−0.124	0.232	−0.533	120.990	0.595				
	attribution-internal stable^1^	−23.309	4.689	−4.971	108.205	< 0.001				
	attribution-external^1^	−4.325	5.046	−0.857	121.095	0.393				
	attribution-internal unstable^1^	−10.892	5.472	−1.991	139.338	0.048				
	lifetime victimization (JVQ)	−0.852	0.417	−2.045	121.578	0.043				
	psychopathology	−0.131	0.172	−0.764	121.505	0.446				
	school day	0.902	0.859	1.051	3131.041	0.293				
	location	−0.703	1.459	−0.482	100.766	0.631				
Model: Social Acceptance	Intercept	104.337	16.600	6.285	119.065	< 0.001	194.195	5.128	37.868	< 0.001
	sex (0)	−2.134	4.454	−0.479	108.300	0.633				
	group (0)	−11.147	4.017	−2.775	113.244	0.006				
	age	0.105	0.239	0.442	122.398	0.660				
	attribution-internal stable^1^	−38.861	5.217	−7.448	167.218	< 0.001				
	attribution-external^1^	−15.733	5.551	−2.834	172.159	0.005				
	attribution-internal unstable^1^	−20.654	5.949	−3.472	181.771	< 0.001				
	lifetime victimization (JVQ)	−0.967	0.424	−2.281	116.702	0.024				
	psychopathology	−1.579	0.174	−0.907	118.224	0.366				
	school day	−0.937	0.718	−1.305	3102.941	0.192				
	location	1.531	1.252	1.223	71.452	0.223				

*JVQ* Juvenile Victimization Questionnaire,^1^ Compared with no victimization experience, *β * standardized regression coefficient, *SE* standard error, *t* *t*-value, *df* degrees of freedom, *p* significance level, *Wald-Z* Wald test statistic

#### Momentary energetic arousal

Surprisingly, everyday victimization experiences and their attributions did not impact arousal state (estimated marginal means: internal stable attribution = 64.7 (*SE* = 4.7), external unstable attribution = 70.8 (*SE* = 5.1), internal unstable attribution = 63.1 (*SE* = 5.7), all pairwise comparisons non-significant). Fixed effect analyses revealed that arousal was only related to location (significant fixed effect: std. *β* = 4.994, *p* = 0.005), meaning that children and adolescents following their usual school routine reported higher arousal than children and adolescents during holidays or weekends. In addition, significant random effects for location (*p* = 0.007) indicated between-subject variability.

#### Momentary calmness

Lifetime victimization, group and attributions for victimization significantly predicted momentary calmness. The fixed effects revealed a significant relationship between lifetime victimization and calmness (*std. β* = −0.852, *p* = 0.043); indicating that higher levels of lifetime victimization were associated with lower levels of calmness. Moreover, the group variable (OOHC or BF) showed a negative relation with calmness, indicating that OOHC reported lower levels of calmness (*std. β* = −10.18, *p* = 0.01). Age, sex, location, psychopathology, school day, and attribution did not influence calmness. Significant random effects for the dependent variable calmness were found for attribution (*p* = 0.003) and location (*p* < 0.001). Estimated marginal means are displayed in Fig. 3. Here, pairwise comparisons between internal stable and external unstable attribution showed significant differences (*p* = 0.004) while internal stable and internal unstable attribution (*p* = 0.075), as well as internal unstable and external unstable attribution (*p* = 0.362), showed no effects.

#### Momentary social acceptance

Momentary feelings of social acceptance were predicted by lifetime victimization experiences (*std. β* = −0.967, *p* = 0.02), by group (*stand. β* = −11.14, *p* = 0.006) and by attribution of victimization experiences in the fixed effect analyses. This means that individuals who experienced more lifetime victimization felt less socially accepted. Furthermore, the OOHC group reported lower levels of social acceptance. With respect to attribution styles, a significant difference was observed for internal stable attribution (*stand. β* = −38.861, *p* < 0.001) and internal unstable attribution (*stand. β* = −20.655, *p* < 0.001) compared to the reference point (no victimization). Additionally, a difference was found between no victimization and external unstable attribution (*stand. β* = −15.732, *p* < 0.005). Age, sex, location, psychopathology, school day, and attribution did not influence social acceptance levels. Significant random effects were detected for attributions (*p* < 0.001) and locations (*p* = 0.002). The estimated marginal means for interpretation, with the dependent variable social acceptance, are displayed in Fig. 3. Pairwise comparisons revealed that those with an external unstable attribution reported significantly higher social acceptance compared to those with an internal stable attribution (*p* = 0.002) and internal unstable attribution (*p* = 0.017). However, no difference was found between both internal attributions (*p* = 0.526).

## Discussion and conclusion

The present study was the first comprehensive investigation using EMA to capture the daily experiences of victimization among participants living with their BF families and those in OOHC. Results highlight both the potential and challenges of using EMA in this context. Below, we discuss the findings in relation to each hypothesis, interpreting results in light of theory and prior research, and identifying limitations and directions for future work.

Hypothesis 1 (increased lifetime and everyday victimization in OOHC vs. BF) was partially supported by our findings. Participants in OOHC indeed reported significantly more lifetime victimization according to parental and self-reports in the JVQ, aligning with extensive literature showing elevated exposure to early trauma and adversity in OOHC populations [2–5]. However, while they reported more everyday victimization in questionnaires, this difference did not reach statistical significance during the 14-day EMA period, possibly due to low overall event frequency and limited power. This has important implications for designing future EMA studies to capture real-life victimization (see below). Importantly, participants in the OOHC group reported being ignored or rejected by friends more often than those in the BF group, highlighting the need to incorporate these more subtle peer experiences into diagnostic assessments in OOHC, particularly given their important role as risk factors for psychological distress among adolescents.

Most commonly, victimization took place at school and was perpetrated by classmates, followed by friends. However, this finding contrasts with the suggestion that children experiencing higher maltreatment are exposed to riskier environments [13]. Additionally, Magallón-Neri et al., [14] noted that highly victimized children spent more time away from home and engaged more in sports, but we did not find increased victimization in those settings. These findings may not contradict each other, as Magallón-Neri et al. [14] did not assess daily victimization with contextual factors. Their results could suggest a coping strategy where highly victimized children seek safer environments during their free time, like sports activities or friends outside of school. Furthermore, our results indicate that attending school is associated with an increased risk of physical and relational victimization, which align with many reports from different countries worldwide [27]. Based on these findings, school-based prevention strategies to reduce victimization might be particularly promising for high-risk youth. However, three meta-analyses of school-based bullying prevention programs have shown a limited impact on reducing bullying [28–30]. As stated by Hong and Espelange [27], the complexity of factors contributing to bullying and peer victimization is often not fully addressed by school- or community-based programs designed to prevent victimization. EMA can serve as a useful tool for evaluating the efficacy of such interventions in real-time (see below).

Hypothesis 2 (psychopathology and lifetime victimization predict everyday victimization) was only partially supported by our findings. As predicted, higher levels of psychopathology were associated with more frequent everyday victimization experiences. This supports theories suggesting that emotional dysregulation and behavioral difficulties can increase vulnerability to negative peer interactions [10, 12]. Additionally, being in OOHC itself was a significant predictor of victimization while, unexpectedly, lifetime victimization experiences were not. The latter finding contrasts with prior research indicating that early maltreatment increases the risk of revictimization [2–5, 7]. One explanation could be that the short two-week EMA window did not adequately capture victimization experiences. Alternatively, the cumulative risk associated with OOHC status may have overshadowed specific contributions from past victimization, especially given potential underreporting or limited recall of early abuse experiences in this specific group [31]. Additionally, the stigma associated with living in OOHC [32] may have further increased the risk of everyday social exclusion. Taken together, these results highlight the importance of assessing current psychopathology and living context (e.g., OOHC) when evaluating victimization risk. Lifetime experiences, while impactful, may exert more indirect effects through their impact on current functioning rather than directly increasing daily victimization.

Our third hypothesis (victimization experiences negatively impact affect and social acceptance) was strongly supported by our findings. Lifetime victimization experiences significantly predicted lower momentary emotional valence, reduced calmness, and diminished feelings of social acceptance, consistent with the bio-psycho-social model of vulnerability [10] and prior studies on affective instability following trauma [16]. Our findings highlight the need to consider both, within- and between-person variability when studying affective dynamics in real-world contexts. Consistent with previous research indicating that bullying victimization contributes to lower mood levels during adolescence [33], our findings support these associations and also confirm a lasting emotional and social impact of early victimization experiences. However, results should be interpreted with caution, as the data are correlational and do not allow for causal conclusions.

Critically, internal attribution styles emerged as robust predictors of lower valence and social acceptance. Youth who interpreted victimization as stemming from internal and stable causes (e.g., “it’s my fault”) experienced worse mood and felt more excluded. This supports attribution theory [11, 12], suggesting that maladaptive cognitive appraisals amplify the emotional burden of victimization and may contribute to a cycle of self-blame and social withdrawal. Interestingly, victimization did not significantly affect energetic arousal, challenging assumptions from trauma models that emphasize heightened arousal as a key marker of dysregulation [10]. This might reflect limitations in self-reported arousal or the moderating role of emotion regulation strategies. Alternatively, the emotional toll of victimization in these youth may manifest more in affective and interpersonal distress than in physiological hyperarousal. Taken together, these findings point to attributional style as a crucial, modifiable risk factor in the pathway from early trauma to emotional difficulties. Interventions that foster adaptive reappraisal strategies could mitigate the emotional and social impact of victimization, especially in OOHC populations.

It is also important to note that no substantial group differences were observed in valence, calmness, or arousal. This lack of difference may indicate significant variability in these emotional states among individuals over time, possibly masking effects between groups. These results emphasize the necessity of accounting for both within- and between-person variability when examining affective dynamics in real-world situations.

### Implications for EMA study designs on daily victimization experiences

Our findings offer important insights for the design of future EMA studies on youth victimization. First, the low event frequency observed over the 14-day period highlights the need for longer sampling windows, potentially one month or more, to capture a broader range of relevant experiences, especially in community or low-incidence samples. The relatively low overall number of reported victimization experiences has limited the statistical power and generalizability of the results in the current study. Second, a substantial proportion of participants, particularly in the OOHC group, did not answer follow-up questions regarding the perpetrators and locations (missing data: OOHC = 50%; BF = 23%). Thus, the high rate of missing contextual data points to a need for simplified prompts or adaptive questioning formats to reduce participant fatigue and improve data completeness.

Third, compliance and data quality could be further enhanced through the use of tailored engagement strategies (e.g., gamification, brief daily incentives). Fourth, the current study relied solely on self-reported affective states; integrating physiological measures (e.g., heart rate, skin conductance) via wearable sensors would allow for a richer, multimodal picture of emotional responses to victimization. Finally, future EMA research could also incorporate follow-up qualitative interviews to help contextualize real-time reports, especially in vulnerable populations that may underreport or misinterpret experiences.

Together, these design improvements could increase EMA studies'reliability, ecological validity, and interpretability, and position this method not only as a powerful tool in identifying but also for intervening on victimization risk in daily life. For example, EMA enables the continuous monitoring of fluctuations in mood, social interactions, and context (e.g., location) throughout the day, providing a nuanced, real-time understanding of individual experiences. By temporally linking these dynamic variables to instances of victimization, EMA can help to identify high-risk patterns or contexts in which victimization is more likely to occur. This approach not only facilitates the development of more targeted interventions but also offers a unique opportunity to evaluate their effectiveness in real-life settings.

### Limitations

Several limitations must be considered. The short EMA duration may have limited our ability to observe episodic or context-specific victimization patterns. Missing data, especially in contextual fields, restricts the depth and interpretability of our analyses. Compliance rates were moderate and lower than typical EMA benchmarks, potentially due to assessment burden. Moreover, the JVQ does not isolate early maltreatment, which limits our ability to pinpoint the developmental timing of adverse events. All emotional data were self-reported, and the absence of physiological markers limits our ability to draw conclusions about arousal or stress responses. Finally, the current study design restricted the assessment of further important risk and protective factors, such as family dynamics or temperament, that may influence both victimization and emotional states. Additionally, while age was considered a covariate in our analyses, developmental differences may still have affected the observed patterns. Those limitations highlight the importance of replication with larger samples and possibly more extended assessment periods to improve the reliability and generalizability of findings.

## Conclusion

This study contributes to our understanding of how victimization is experienced and interpreted in daily life, particularly among youth in OOHC. Findings confirm the importance of current psychopathology and cognitive appraisals in predicting victimization and its emotional toll. Internal attributions for victimization appear especially detrimental, suggesting a promising target for cognitive-behavioral interventions. Although methodological limitations exist, this work illustrates the potential of EMA to uncover real-time mechanisms of revictimization and inform future research and prevention efforts.

## Supplementary Information

Below is the link to the electronic supplementary material.

Supplementary file1 (DOCX 31 KB)

## Data Availability

The data will be made publicly available in an anonymized form after the project is completed and potentially indirect identifiers that could lead to a possible re-identification of critical personal data in the German out-of-home care system are removed. Currently, data access requests can be sent to the Child and Adolescent Psychiatry, University Hospital RWTH Aachen: (KJP-Sekretariat@ukaachen.de).

## Abbreviations

BF: Biological Families
OOHC: Out-Of-Home Care
EMA: Ecological Momentary Assessment
