# Supplementary material for: Understanding everyday victimization experiences in vulnerable youth: an ecological momentary assessment approach
Source: Eur Child Adolesc Psychiatry. 2025 Jul 31;35(1):177–89. doi: 10.1007/s00787-025-02829-z (PMC12917016; doi:10.1007/s00787-025-02829-z)
Supplement: Supplementary file 1 — Supplementary file1 (DOCX 31 KB) [file 787_2025_2829_MOESM1_ESM.docx]

Supplementary material

Table 4: EMA Victimization Experience per Question

| Victimization Experience | Number of Events | OOHC (N 68)  (Frequency/Percentage/Cum. Percentage) | BF (N 89)  (Frequency/Percentage/Cum. Percentage) | test for equality of proportions  (df/ X^2^/FDR-adjusted p-value) |
| --- | --- | --- | --- | --- |
| 1 : you were forced by someone to do something you didn't want to do? | 0  1  2-4  5+ | 55 /80.9/80.9  5/7.4/97.1  6/8.8/89.7  2/2.9/100 | 83/93.3/93.3  5/5.6/98.9  1/1.1/100  0/0/0 | 3/ 7.1/.20 |
| 2: someone has physically approached or harassed you against your will (e.g. touched you or stalked you)? | 0  1  2-4  5+ | 61/89.7/89.7  4/5.9/95.6  3/4.4/100  0/0/100 | 85/95.5/95.5  2/2.2/97.8  2/2.2/100  0/0/100 | 3/2.6/.53 |
| 3 : someone has insulted you, threatened you or called you names? | 0  1  2-4  5+ | 49/72.1/72.1  8/11.8/83.8  9/13.2/97.1  2/2.9/100 | 75/84.3/84.3  11/12.4/96.6  1/1.1/97.8  2/2.2/100 | 4/11.3/.14 |
| 4 : someone has intentionally excluded you (e.g. from meetings, activities or groups)? | 0  1  2-4  5+ | 62/91.2/91.2  3/4.4/95.6  2/2.9/98.5  1/1.5/100 | 83/93.3/93.3  2/2.4/96.6  3/3.4/100  0/0/100 | 3/.19/.98 |
| 5: someone has spread lies or rumours about you? | 0  1  2-4  5+ | 59/96.6/96.6  3/3.4/100  0/0/100  0/0/100 | 87/97.8/97.8  1/1.1/98.9  1/1.1/100  0/0/100 | 3/7.3/.91 |
| 6 : someone specifically ignored you or no longer wanted to be friends with you? | 0  1  2-4  5+ | 51/75/75  12/17.6/92.6  4/5.9/98.5  1/1.5/100 | 86/96.6/96.6  3/3.4/100  0/0/100  0/0/100 | 2/12.2/.00** |
| 7 : you have been ridiculed by a person or a group? | 0  1  2-4  5+ | 69/86.8/86.8  4/5.9/92.6  3/4.4/97.1  2/2.9/100 | 81/91/91  7/7.9/98.9  1/1.1/100  0/0/100 | 3/2.3/.52 |
| 8 : someone has taken something from you or broken something on purpose? | 0  1  2-4  5+ | 61/89.7/89.7  4/5.9/95.6  3/4.4/100  0/0/100 | 80/89.9/89.9  6/6.7/96.6  3/3.4/100  0/0/100 | 3/.17/.98 |
| 9 : someone has hit you, pushed you, beaten you or physically assaulted you in any other way? | 0  1  2-4  5+ | 63/92.6/92.6  3/4.4/97.1  2/2.9/100  0/0/100 | 83/93.3/93.3  5/5.6/98.9  1/1.1/100  0/0/100 | 3/.96/.91 |
| 10 : someone has published your private messages or spreaded them further against your will? | 0  1  2-4  5+ | 67/98.5/98.5  1/1.5/100  0/0/100  0/0/100 | 88/98.9/98.9  1/1.1/100  0/0/100  0/0/100 | 2/.06/.97 |
| 11 : someone has taken, posted or shared photos of you on the internet without your consent? | 0  1  2-4  5+ | 64/94.1794.1  4/5.9/100  0/0/100  0/0/100 | 85/95.5/95.5  4/4.5/100  0/0/100  0/0/100 | 2/.19/.95 |
| 12 : you have received threatening or aggressive messages in general via the internet or your mobile phone (e.g. also right-wing extremist or pornographic content)? | 0  1  2-4  5+ | 64/94.1/94.1  3/4.4/98.5  1/1.5/100  0/0/100 | 85/95.5/95.5  4/4.5/100  0/0/100  0/0/100 | 2/.01/.99 |
| 13 : you did not feel loved by your parents? | 0  1  2-4  5+ | 53/77.9/77.9  10/14.7/92.6  4/5.9/98.5  1/1.5/100 | 77/86.5/86.5  8/9/95.5  3/3.4/98.9  1/1.1/100 | 4/2.5/.78 |
| 14 : you did not feel supported by your parents? | 0  1  2-4  5+ | 55/80.9/80.9  7/10.3/91.2  6/8.8/100  0/0/100 | 74/83.1/83.1  9/10.1/93.3  5/5.6/98.9  1/1.1/100 | 3/.74/.90 |

_Note: physical victimization = 2, 8, 9; relational victimization: 1, 3, 6, 4, 5, 7; cyber: 10, 11, 12; and parents: 13, 14._

***Figure 4: Locations of Relational Victimization Experiences (EMA)***

_Note: OOHC = Out of home care; Percentages reflect self-reported data_

***Figure 5: Locations of Physical Victimization Experiences (EMA)***

_Note: OOHC = Out of home care; Percentages reflect self-reported data_

***Figure 6:*** ***Perpetrators of Relational Victimization Experiences (EMA)***

_Note: OOHC = Out of home care; Percentages reflect self-reported data_
